# Editorial: New advances in knowledge of the parasite-host relationship during malarial infection

**DOI:** 10.3389/fimmu.2022.1041240

**Published:** 2022-10-11

**Authors:** Letusa Albrecht, Silvia Beatriz Boscardin, Kezia Katiani Scopel

**Affiliations:** ^1^ Apicomplexa Research Laboratory, Carlos Chagas Institute, Oswaldo Cruz Foundation, Curitiba, Brazil; ^2^ Departamento de Parasitologia, Universidade de São Paulo, Instituto de Ciências Biomédicas, Sao Paulo, Brazil; ^3^ Department of Parasitology, Microbiology and Immunology, Juiz de Fora Federal University, Juiz de Fora, Brazil

**Keywords:** malaria, parasite-host relationship, surface antigens, malaria in pregnancy, avian malaria, microbiota

The interaction between a pathogen and its host can lead to health or disease. Malaria is caused by different *Plasmodium* species, and even if most attention is directed to those species that cause human malaria, *Plasmodium* parasites can infect several other vertebrates, including birds. This Special Research Topic of Frontiers in Immunology brings original, mini-reviews, perspectives, and hypothesis and theory articles that explore the parasite-host relationship during malaria infection in humans and birds.

In humans, malaria is mainly caused by *P. falciparum* and predominantly affects children in the first years of life and pregnant women. The course of infection depends on the interaction between the host’s immune response and the parasite virulence. In severe cases, a dysregulated immune response is present. Furthermore, an interaction between the parasite and the endothelium could lead to endothelial activation. Even if diagnostics of malaria are quite well established, the prognosis of severe and fatal malaria cases is very challenging. A host factor that could be useful in this prediction is the soluble urokinase-type plasminogen activate receptor (suPAR), as reviewed by Stefanova et al..

Parasite surface antigens, such as PfEMP1, STEVOR, and RIFIN proteins, interact with the host and are important for parasite evasion. In this Research Topic, Sakoguchi and Arase reviewed recent discoveries of how surface erythrocytic antigens of *P. falciparum* can interact with human inhibitory immune receptors.

The interaction between the parasite and the host was also discussed in the context of malaria in pregnancy (MiP). MiP can be a severe disease and various factors can be important for the final outcome. Despite that, the molecular mechanisms involved in gestational malaria are still not clear. Placental autophagy is modulated primarily by immune signals and inflammation and is dysregulated in pregnant women infected with *P. falciparum*. In this special Research Topic, Barateiro et al. wrote a Perspective article, reviewing the main findings concerning placental autophagy in malaria, and hypothesized that placental autophagy plays a cytoprotective role during infection in pregnancy.

During the erythrocytic *Plasmodium* cycle, heme is released upon red blood cell rupture, which can lead to inflammation and tissue damage. The main mechanism of heme catalysis is through the activity of the enzyme heme oxygenase 1 (HO-1). HO-1 plays a dual role: while it is associated with successful pregnancy; in inflammatory conditions, it can induce iron overload and cell death. In this Research Topic, Cariaco et al. investigated the role of HO-1 in gestational malaria using a murine model. They found that HO-1 expression and activity are enhanced in *Plasmodium* infection during early pregnancy. They also showed that ZnPPIX treatment decreased the ability of HO-1 to develop its deleterious effect when in excess.

Host-microbiota interactions can lead to resistance or susceptibility to an infection. In this Research Topic a ‘Hypothesis and Theory’ paper discusses the use of bird-malaria-microbiota system to understand the implications of gut microbiota diversity to anti-α-Gal (Palinauskas et al.). Microbiota was also evaluated in the context of an avian malaria vaccine (Aželytė et al.).

Understanding how the parasite interacts with the host and how the host responds to this interaction is fundamental for developing new tools for malaria interventions. In this Research Topic, aspects of the parasite-host relationship were discussed.

## Author contributions

All authors listed have made a substantial, direct, and intellectual contribution to the work and approved it for publication.

## Conflict of interest

The authors declare that the research was conducted in the absence of any commercial or financial relationships that could be construed as a potential conflict of interest.

## Publisher’s note

All claims expressed in this article are solely those of the authors and do not necessarily represent those of their affiliated organizations, or those of the publisher, the editors and the reviewers. Any product that may be evaluated in this article, or claim that may be made by its manufacturer, is not guaranteed or endorsed by the publisher.

